# Investigation of half metallic properties of Tl_2_Mo(Cl/Br)_6_ double perovskites for spintronic devices

**DOI:** 10.1039/d4ra01759e

**Published:** 2024-05-24

**Authors:** M. Ammar Yasir, Ghulam M. Mustafa, Bisma Younas, N. A. Noor, Mehdi Ali, Sadia Nazir, Ahmed Z. Dewidar, Hosam O. Elansary

**Affiliations:** a Department of Physics, RIPHAH International University Campus Lahore Pakistan; b Department of Physics, Division of Science and Technology, University of Education Lahore Punjab 54770 Pakistan; c Department of Physics, University of Lahore Lahore 53700 Pakistan Sadiamalik.chep@gmail.com; d The University of Electro-Communications Tokyo Japan; e Prince Sultan Bin Abdulaziz International Prize for Water Chair, Prince Sultan Institute for Environmental, Water and Desert Research, King Saud University Riyadh 11451 Saudi Arabia helansary@ksu.edu.sa; f Department of Agricultural Engineering, College of Food and Agriculture Sciences, King Saud University Riyadh 11451 Saudi Arabia; g Plant Production Department, College of Food & Agriculture Sciences, King Saud University Riyadh 11451 Saudi Arabia

## Abstract

The manipulation of electronic device characteristics through electron spin represents a burgeoning frontier in technological advancement. Investigation of magnetic and transport attributes of the Tl_2_Mo(Cl/Br)_6_ double perovskite was performed using Wien2k and BoltzTraP code. When the energy states between ferromagnetic and antiferromagnetic conditions are compared, it is evident that the ferromagnetic state exhibits lower energy levels. Overcoming stability challenges within the ferromagnetic state is achieved through the manipulation of negative Δ*H*_f_ within the cubic state. The analysis of the half metallicity character involves an analysis of band structure (BS) and DOS, elucidating its mechanism through PDOS using double exchange model p–d hybridization. The verification of 100% spin polarization is confirmed through factors such as spin polarization and the integer value of the total magnetic moment. Furthermore, the thermoelectric response, as indicated by the ratios of thermal-electrical conductivity and *ZT*, underscores the promising applications of these compounds in thermoelectric device applications.

## Abstract 2

The manipulation of electronic device characteristics through electron spin represents a burgeoning frontier in technological advancement.

## Graphical abstract

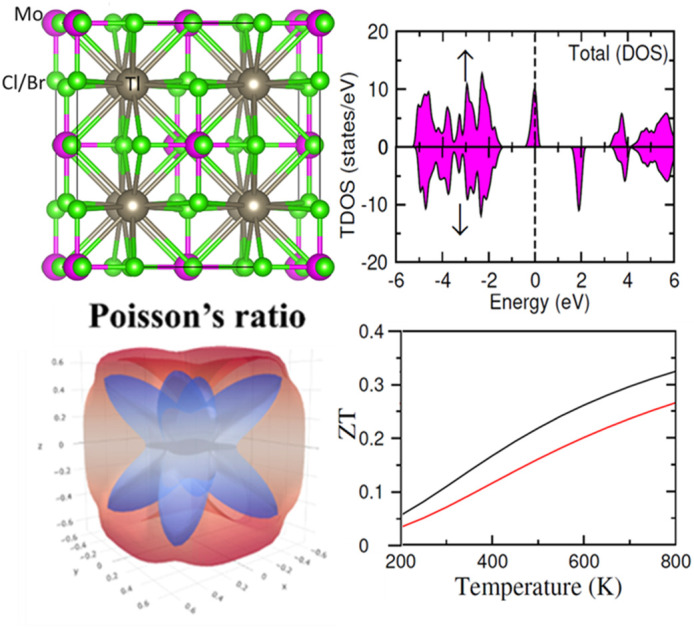

## 1. Introduction

Spintronics, a new research area at the intersection of physics and electronics, has become particularly interesting owing to its possible influence on the development of novel types of electronic devices based on electron spin (their intrinsic property).^1^ Unlike in traditional electronics, which mainly rely on electron charges for data handling and storage, spintronics employs the electron's spin degree of freedom, which results in the appearance of electronic devices with novel and enhanced functionalities.^2^ Spintronics is actually a branch of electronics based on one of the fundamental concepts, namely half metallicity, which is a special property of materials characterized by the fact that they have different conductive properties for electrons with different spin orientations. The interest in half-metallicity lies in its spintronic features, where one spin is conducting while the other spin is insulating.^3–5^ This property provides completely novel capabilities for controlling and manipulating the magnetic moment of itinerant electrons and opens new avenues for utilizing this spin dependence in various devices. The investigation of half-metallicity in spintronics has opened up new avenues for the fabrication of spin-based electronic devices such as spin valves, magnetic tunnel junctions and spin transistors, which depend on differentiating and manipulating the spin-polarized currents.^6^ In addition, the design and production of half-metallic materials in spintronics directly affect spin-dependent phenomena such as colossal magneto-resistance and tunnel magneto-resistance, which are limiting factors in high-density magnetic data storage and spintronic memory devices.^7^ Researchers have targeted the inherent traits of the half metallic substances and intend to outdo the outmoded limitations in electronic devices in terms of power consumption, speed and scalability to ready the juncture for the forthcoming spintronic innovations. Concurrently, the quest for new materials has made this discovery of double perovskites, a very diverse group of compounds famed for their versatile properties.^8^ In this scenario, the origins of spintronics, half-metallicity, and double perovskites are of great importance, affecting material design and technological innovation.

Besides the emergence of half-metallic materials, the class of compounds known as double perovskite has gained attention because of its unique properties of electronic and magnetic capability due to its complex crystal structure and various cationic configurations. The perovskite structure is characterized by a simplified addressing by the general formula. A_2_BB′O_6_ can be regarded as a versatile framework for the investigation of a broad spectrum of physical phenomena and the design of material properties through composition engineering.^9^ Double perovskites, a subgroup of perovskite materials featuring the simultaneous occupation of two cations at the B site, display various high-order electronic structures with a rich phase space that can lead to numerous electronic transport characteristics. Such engineering would yield a structure with mutable electronic band structures through approaches including, but not limited to, band engineering, strain engineering, and doping. In addition, the functionality of the transition metal nodes of the perovskite lattice leads to significant phase space, and the existence of electronic phenomena, such as colossal magnetoresistance and multiferroicity, is possible. Additionally, the intriguing interplay between phonon properties and electronic states in double perovskite material provides a way to explore novel phenomena that may enhance the properties of the thermoelectric *ZT*. The essence of the thermoelectric function of double perovskites is the complex relationship between the electronic structure, charge transport, and phonon scattering mechanisms.^10^ The band engineering possibilities from the dual-cation arrangement ensure that the electronic states can be manipulated, and their mobility is improved, thereby, in the process, boosting the thermoelectric efficiencies.^11^ Furthermore, particular d-electrons of transition metal ions and conducting oxygen p-orbitals also play a role in the complex electronic behavior of the materials; this means that such compounds have ideal properties for further development of thermoelectric properties.^12^

Numerous researchers have allocated their findings specifically to studying the double perovskite group due to the intriguing thermoelectric properties of this material. Mahmood *et al.* studied the ferromagnetic thermoelectric response of K_2_Z(Cl/Br)_6_ (Z = Ta, W, Re), which is related to its application in spintronics and energy. Their investigation delivered an in-depth description of the features of these materials when comparing them in terms of their different possible uses in future technological developments. Mahmood *et al.*'s findings provide a significant impetus for research in materials for spintronic and energy applications.^13^ Alburaih *et al.* presented a theoretical investigation, relying thoroughly on DFT calculations, of vacancy-ordered single-crystalline K_2_TcZ_6_ (Z = Cl, Br) systems searching owing to their suitable characteristics for spintronic applications. Their work focused on the electronic structure and magnetic response in the studied composition and provided a basis for the development of new spintronic devices and data storage.^14^ In the research by Mahmood *et al.* (2022), the impact of 5d electrons on half metallic ferromagnetic behavior and conduction of Cs_2_Z(Cl/Br)_6_ (Z = Os, Ir) for devices in spintronics was explored.^15^ The study featured a comprehensive analysis of the behavior of these materials, which served as the basis for the development of novel spintronic devices. The authors explored the relationship between 5d electrons and their proprieties in detail, providing contributions that can be valuable for materials. Their findings emphasize that the Cs_2_Z(Cl/Br)_6_ (Z = Os, Ir) compound might play a key role in the development of spintronics, which again makes them theoretically critical for future science and technology. The molecules Mo and Tl provide remarkably strong spins used in the devices to store and process information using spins. As far as the tunability of Tl and Mo and the possibility of application in devices with the magnetization effect are concerned, magnetronics could be a crucial field. With our calculations, we are confident that Tl_2_Mo(Cl/Br)_6_ will definitely be the best composition in spin-based technology. Perovskites with halide composition exhibit applications in several spintronic gadgets, including magnetic memory, spin valves, and spin orbit applications due to the unique magnetic and electrical tunability of the combination. Spintronics has been performing very well, and further advancements in this area will potentially command various possibilities and highly adaptive spintronic device functionalities. The principal objective of the present investigation is to acquire a more comprehensive understanding of the characteristics of Tl_2_Mo(Cl/Br)_6_, covering aspects such as structural, electronic, elastic, magnetic, and transport properties.

## 2. Computational methodology

The stability of a material's structure is crucial because it governs numerous physical attributes. In this study, Tl_2_Mo(Cl/Br)_6_ was analyzed using a DFT-based FP-LAPW approach implemented using Wien2k code.^16^ Ground-state energies were computed using the PBE-sol approximation, with adjustments made to accurately determine bandgaps through the mBJ potential.^17^ Notably, PBE-sol, a modified version of PBE, incorporates different constraints, offering improved accuracy, particularly for heavy metal systems related to PBE. The TB-mBJ potential, known for its precision akin to HSE06, was employed for electronic BS and DOS computations, alongside consideration of spin–orbit coupling.^18,19^ To ensure convergence, a *K*-mesh of 2000 *k*-points (12 × 12 × 12) was utilized and later increased to 20 × 20 × 20 for thermoelectric calculations. Parameters such as (muffin-tin radius) *R*_MT_ × ***K***_max_ (***K***-vector), 
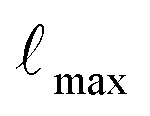
 (angular momentum vector) and *G*_max_ (Gaussian factor) were carefully selected for accuracy, with computed results converging to the order of 10^−3^ mRy. Transport response was estimated utilizing the BoltzTrap code, providing an inclusive analysis of the material's properties.^20^

## 3. Results and discussion

### 3.1 Structural analysis

**Fig. 1:**
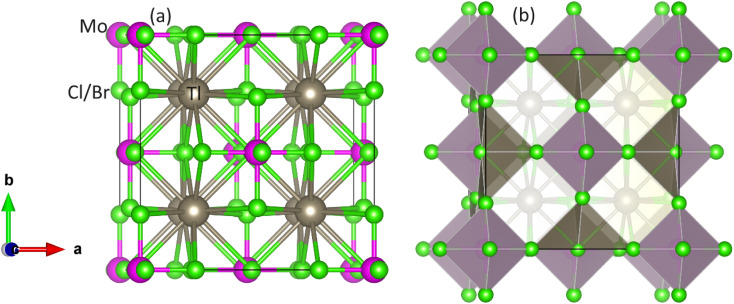
Unit cell of DP Tl_2_Mo(Cl/Br)_6_: (a) ball-stick and (b) polyhedral model. Gray, pink and green balls show Tl, Mo, and Cl/Br atoms, respectively.

**Fig. 2:**
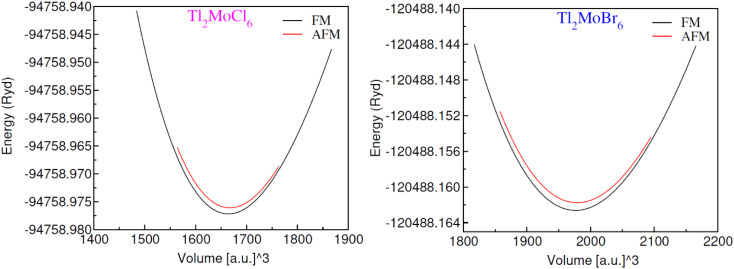
Energy against volume plot for Tl_2_MoCl_6_ and Tl_2_MoBr_6_ in ferromagnetic (FM) and anti-ferromagnetic (AFM) states.

The optimization of energy release from compounds with *Fm*3̄*m* (no. 225) space group in cubic phase and determination of ground-state parameters were conducted using the Birch–Murnaghan equation of states (Table 1).^21^Fig. 1 illustrates unit cells in both ball-stick (left) and polyhedral (right) formats, showcasing atomic arrangement and geometric configuration, respectively. Within MoCl/Br_6_ octahedra, the Tl atom fills vacancies, which exhibit 12-fold coordination with Cl/Br atoms, resulting in individual octahedra being distinct from the others. The Mo atoms occupies the center of the octahedra, encircled by six Cl/Br atoms, while the Mo atoms are also present at the face centers and corners.^22^ Wyckoff positions for Tl (8c), Mo (4a), and Cl/Br (24e) were utilized for structure generation and optimization. Through the replacement of Cl with Br, atomic radii increase, consequently enlarging the lattice constant in the range of 9.95–10.54 Å and inter-atomic distance, thus reducing material density and solidness, and leading to a decrease in bulk moduli (*B*) from 43.75–35.95 GPa.^23^ The same type of variation in the lattice constant has also been observed by Mahmood *et al.* in Cs_2_ReCl/Br_6_ double perovskites.^11^ The considerable bulk modulus of Tl_2_MoBr_6_ suggests greater stiffness compared to Tl_2_MoCl_6_. Energy plots against volume in the ferromagnetic (FM) and anti-ferromagnetic (AFM) phases, as displayed in Fig. 2, reveal higher energy release in the FM state, indicating its greater favorability. Furthermore, energy formation was computed to evaluate thermal stability using the following equation:^24^Δ*H*_f_ = *E*_Total_(Tl_*l*_Mo_*m*_(Cl/Br)_*n*_) − *lE*_Tl_ − *mE*_Mo_ − *nE*_Cl/Br_,where *E*_Total_ denotes the total energy of double perovskites; *E*_Tl_, *E*_Mo_, and *E*_Cl/Br_ denote energies of Tl, Mo, and Cl/Br, respectively; *l* denotes the number of Tl; *m* denotes the number of Mo and *n* denotes the number of Cl/Br atoms.^25^ The Δ*H*_f_ for Tl_2_MoCl_6_ is calculated as −1.44 eV. Similarly, for Tl_2_MoBr_6_ Δ*H*_f_ are found to be −1.18 eV for Br. This indicates the thermal stability of the compounds, as evidenced by the negative values of Δ*H*_f_ in the ferromagnetic state. Another crucial aspect of FM materials in spintronic device applications is the ability to maintain ferromagnetism above RT. To determine Curie temperature (*T*_c_), the Heisenberg model *T*_c_ = 2Δ*E*/3*xK*_B_ was employed, where *x* represents the Mo concentration and Δ*E* = *E*_AFM_ − *E*_FM_, as illustrated in Table 2. The computed *T*_c_ for Tl_2_MoCl_6_ is 567 K and for Tl_2_MoBr_6_ is 533 K, indicating that the FM nature is above room temperature (RT).^26^

**Table 1:** Computed lattice parameters, bulk modulus values, ground-state energy differences, Curie temperature (*T*_c_) and formation energy (Δ*H*_f_ (eV)) of DPs Tl_2_Mo(Cl/Br)_6_

Chalcogenides	*a*_o_ (Å)	*B*_0_ (GPa)	Δ*E* = *E*_AFM_ − *E*_FM_	*T*_c_ (K)	Δ*H*_f_
Tl_2_MoCl_6_	9.95	43.75	22.66	567	−1.44
Tl_2_MoBr_6_	10.54	35.95	14.88	533	−1.18

**Table 2:** Computed elastic constants (*C*_11_, *C*_12_, *C*_44_) and computed mechanical parameters for Tl_2_Mo(Cl/Br)_6_

	*C*_11_	*C*_12_	*C*_44_	*B*_0_	*G*	*Y*	*B*_0_/*G*	*υ*	*α*
Tl_2_MoCl_6_	88.09	20.82	13.81	43.24	19.90	51.77	2.17	0.30	0.41
Tl_2_MoBr_6_	71.55	16.13	14.06	34.60	18.51	47.13	1.86	0.27	0.51

### 3.2 Mechanical properties

The material's performance under stress is defined by elastic constants, ensuring a mechanically stable crystal structure.^27^ Cubic symmetric properties rely on three elastic constants (*C*_11_, *C*_12_, and *C*_44_), where Born stability conditions (*C*_11_ − *C*_12_ > 0, *C*_44_ > 0, *C*_11_ + 2*C*_12_ > 0, and *C*_12_ < *B*_0_ < *C*_11_) support mechanical stability, as listed in Table 2.^28^ The *B* parameter, calculated as *B*_0_ = (*C*_11_ + 2*C*_12_)/3, elucidates mechanical behavior, indicating that Tl_2_MoBr_6_ exhibits lower output compared to Tl_2_MoCl_6_, which is consistent with *B* calculated from optimization.^29^ Ductility differentiation between materials was determined by controlling Poisson's (*ν* > 0.26) and Pugh's (*B*_0_/*G* > 1.75) ratio.^30^ When the component number exceeds the cut-off value, it signifies greater deformability than rigidity. The investigated composition's ductility is presented in Table 2. Anisotropy (*A* = 2*C*_44_/(*C*_11_ − *C*_12_)) provides additional insight into directional characteristics, where isotropic materials possess a unit value and anisotropic materials exhibit a value less than unity (Table 2).^31^

### 3.3 Elastic anisotropy

**Fig. 3:**
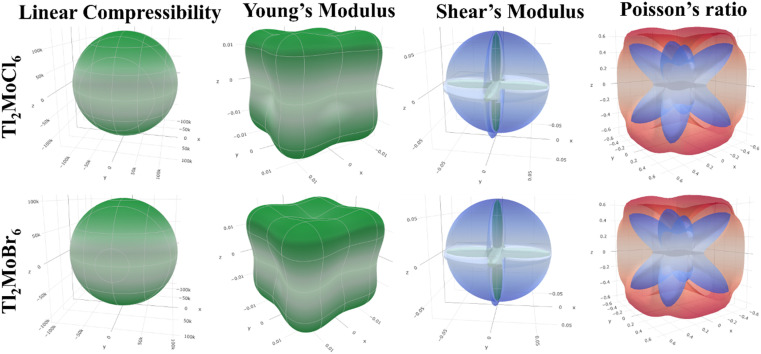
Representation of elastic moduli in 3-D.

Elastic anisotropy represents the significant physical characteristics of a material, indicating variations in physical and chemical properties with directional changes.^32^ Isotropic materials maintain reliable characteristics irrespective of measurement directions, exhibiting identical performance values in all directions. Fig. 3 depicts 3-D surface *c* representations of Youngs (*Y*), shear (*G*) moduli, and Poisson (*ν*) ratio for Tl_2_Mo(Cl/Br)_6_. Isotropic materials manifest spherical 3D surface structures, while deviations from a sphere denote anisotropy, with greater deviations indicating stronger anisotropy.^33,34^ The 3-D figures for Tl_2_MoCl_6_ do not resemble spheres, confirming its anisotropic nature. Further investigation into Tl_2_Mo(Cl/Br)_6_ elastic anisotropy involves examining values such as *β*_max_, *β*_min_, *Y*_max_, *Y*_min_, *G*_max_, *G*_min_, *ν*_max_, and *ν*_min_, along with ratios such as *β*_max_/*β*_min_, *Y*_max_/*Y*_min_, *G*_max_/*G*_min_, and *ν*_max_/*ν*_min_ (Table 3). For an isotropic material, the ratio of the maximum to minimum elastic modulus is equal to 1,^35^ whereas anisotropic materials exhibit ratios different from 1, with higher ratios indicating stronger anisotropy. In the case of Tl_2_MoCl_6_, *β*_max_/*β*_min_ = 1, *Y*_max_/*Y*_min_ = 1.841, *G*_max_/*G*_min_ = 9.742, and *ν*_max_/*ν*_min_ = 0.259, indicating greater anisotropy compared to Tl_2_MoBr_6_. Among the studied allotropes, Tl_2_MoCl_6_ demonstrates the highest anisotropy.^36^

**Table 3:** Maximum and minimum values of elastic moduli and their anisotropy for Tl_2_Mo(Cl/Br)_6_

Parameters	Linear compressibility (*β*) (TPa^−1^)			Young's modulus (*Y*) (GPa)			Shear modulus (*G*) (GPa)			Poisson's ratio (*ν*)		
	*β*_min_	*β*_max_	*A*	*Y*_min_	*Y*_max_	*A*	*G*_min_	*G*_max_	*A*	*ν*_min_	*ν*_max_	*A*
Tl_2_MoCl_6_	129 730	129 730	1	0.01135	0.0209	1.841	0.00743	0.072411	9.742	−0.91325	−0.23635	0.259
Tl_2_MoBr_6_	103 810	103 810	1	0.01397	0.0255	1.821	0.00902	0.071124	7.883	−0.87943	−0.22544	0.256

### 3.4 Electronic properties

**Fig. 4:**
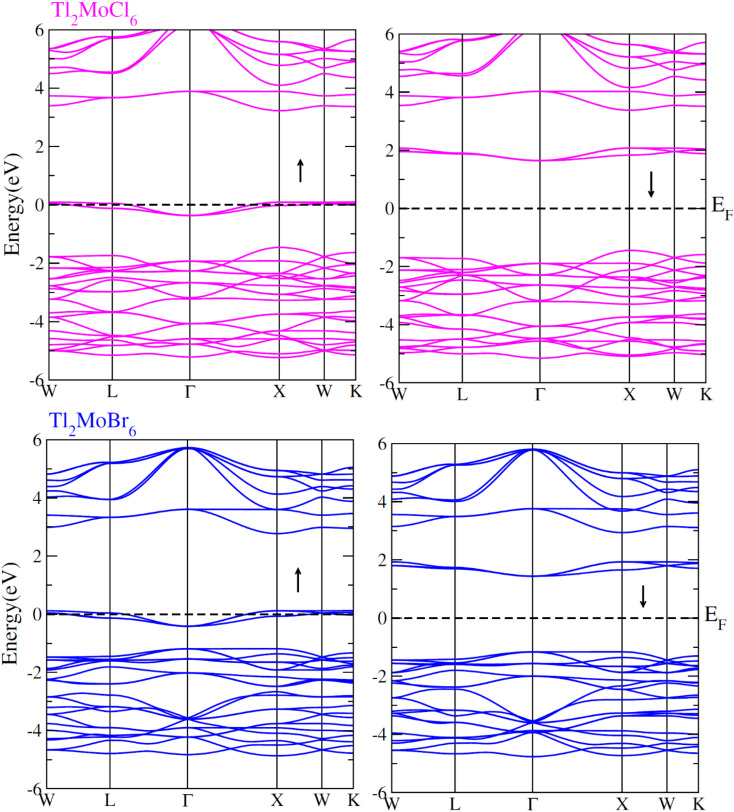
Band structures computed for Tl_2_Mo(Cl/Br)_6_ for up (↑) and down (↓) spin configurations using mBJ potential.

**Fig. 5:**
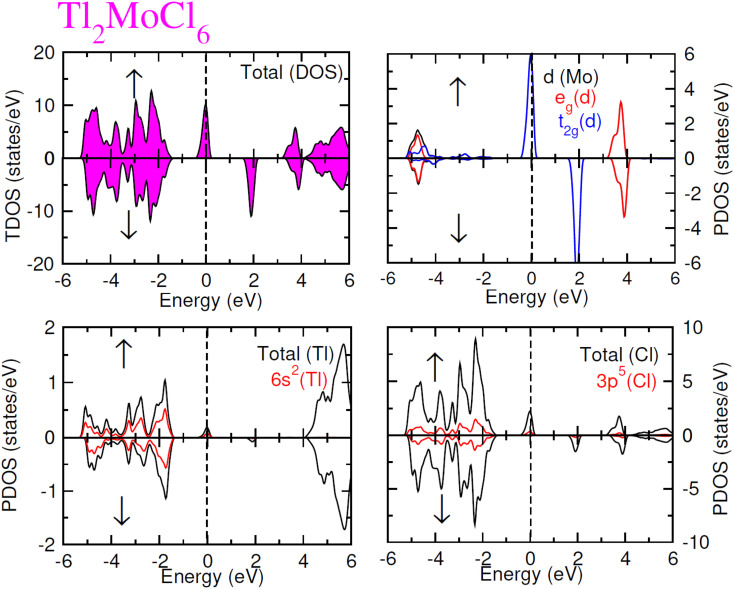
Total and partial DOS for Tl_2_MoCl_6_ with Tl, Mo and Cl atoms in spin up (↑) and down (↓) spin configurations.

**Fig. 6:**
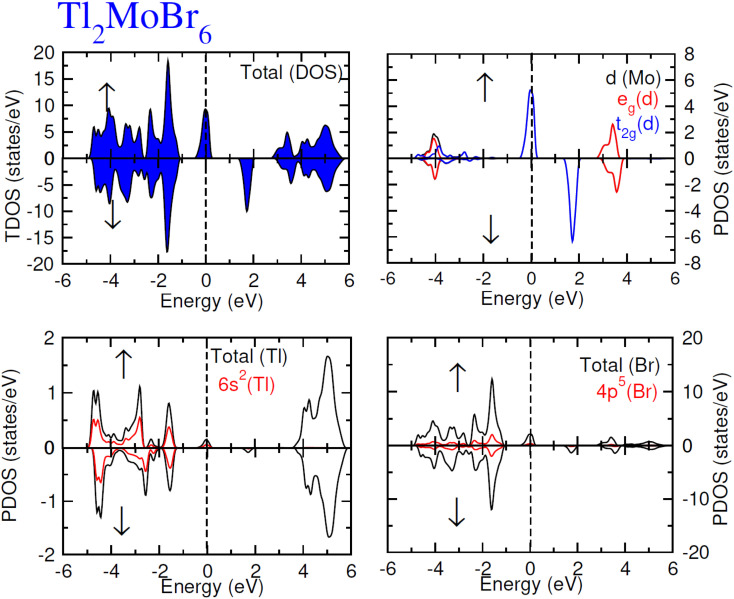
Total and partial DOS for Tl_2_MoBr_6_ with Tl, Mo and Br atoms in spin up (↑) and down (↓) spin configurations.

**Fig. 7:**
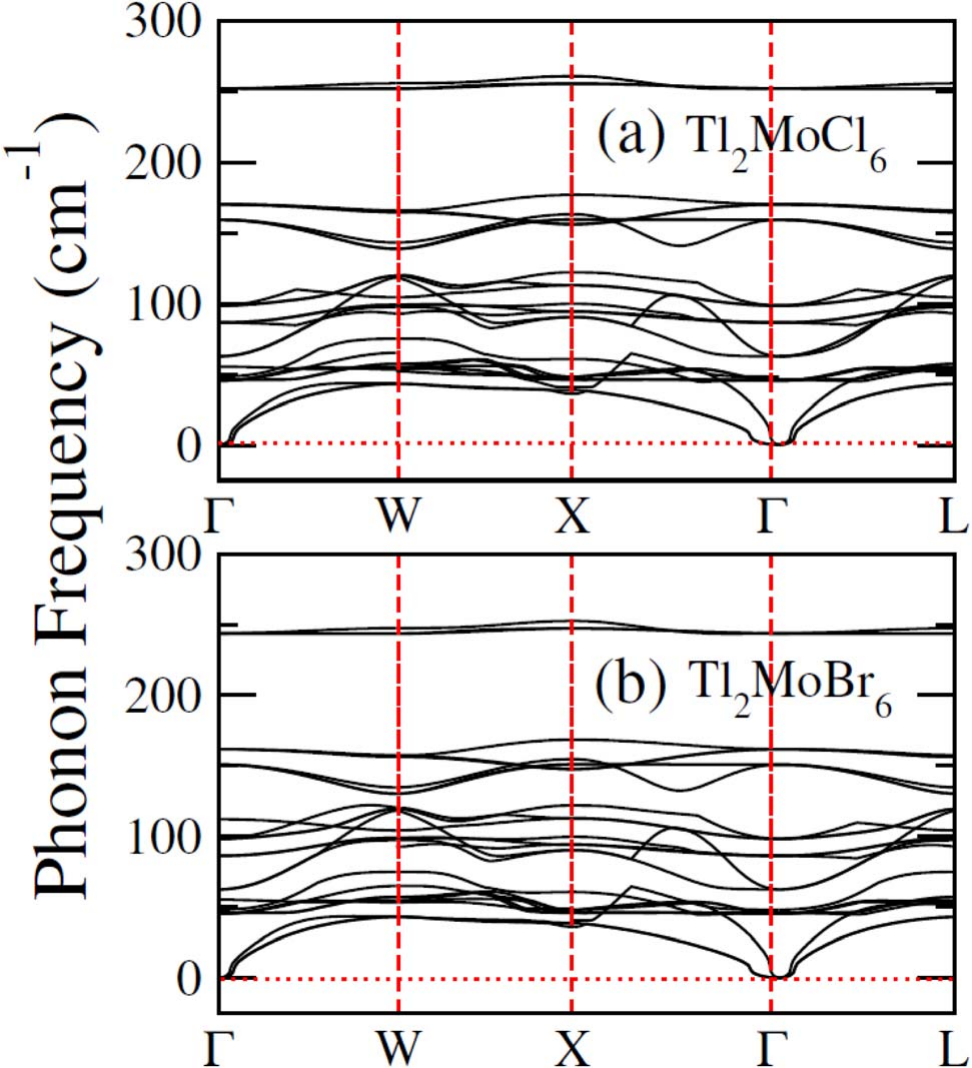
Computed phonon dispersion plot for (a) Tl_2_MoCl_6_ and (b) Tl_2_MoBr_6_.

To elucidate the electronic behavior of Tl_2_Mo(Cl/Br_6_), we generated visual representations of band structures (BS) in Fig. 4, and the density of states (DOS) is depicted in Fig. 5 and 6. The band structures unveil state presence at Fermi level (*E*_f_) within spin-up (↑) configuration, exhibiting characteristics akin to direct bandgap semiconductors. Conversely, within the down (↓) spin channel, the *E*_f_ resides in the band-gap, indicating insulating properties.^37^ This transition of states between the up (↑) and down (↓) configurations underscores the manifestation of the ferromagnetic character. It is imperative to note that achieving complete 100% spin-polarization serves as a fundamental prerequisite for half-metallic FM behavior. Hence, spin-polarization is quantitatively assessed *via* the equation *P* = (*N*_↓_(*E*_F_) − *N*_↑_(*E*_F_))/(*N*_↓_(*E*_F_) + *N*_↑_(*E*_F_)), where *N*_↓_(*E*_F_) denotes the DOS in the down (↓) spin and *N*_↑_(*E*_F_) denotes the DOS at *E*_f_ in the up (↑) spin configuration. Notably, within the up (↑) spin configuration, states are situated precisely at *E*_f_, whereas in the down (↓) spin configuration, the *E*_f_ is notably absent at that position, as depicted in Fig. 5 and 6. Consequently, the investigated halides exhibit complete spin polarization (*P* = 1).^38^ Furthermore, to give an inclusive insight into ferromagnetism, the density of states (DOS) is graphically illustrated in Fig. 5 and 6. An analysis of the total density of states (TDOS) reveals that the up (↑) spin channel exhibits characteristics of direct bandgap semiconductors along the Γ symmetry direction, whereas the down (↓) spin channel demonstrates an insulating nature. Consequently, the interplay between semiconductor and insulator responses facilitates electron exchange, thereby inducing ferromagnetic characteristics. The basic driver of ferromagnetism in Tl_2_MoCl_6_ and Tl_2_MoBr_6_ stems from the individual electronic states of Mo, Tl, and Cl/Br hybridization. Specifically, the d-state of Mo splits into t_2g_ and e_g_ states of Mo upon encountering the octahedral and tetrahedral environments of halide atoms.^39^ The e_g_ state shifts to low energy levels, whereas the t_2g_ state ascends to high energy levels. Among these, the t_2g_ state, comprising d_*xy*_, d_*yz*_, and d_*zx*_ orbitals, exhibits a linear trend and significantly contributes to hybridization, whereas the e_g_ state, comprising d_*z*^2^_ and d_*x*^2^−*y*^2^_ orbitals, displays nonlinear behavior and plays a negligible role in ferromagnetism. The distinction between e_g_ and t_2g_ states is quantified as crystal field energy (Δ*C*_F_ = e_g_ − t_2g_).^40^ To promote ferromagnetic behavior, it is imperative to minimize the crystal field energy through direct shifts of Mo d-states. The direct exchange energy *Δ*_*x*_(d) = *Δ*(d↓) − *Δ*(d↑), representing the energy difference of d states among down (↓) and up (↑) spin configurations, must exceed *C*_F_ energy (*Δ*_*x*_(d) > Δ*C*_F_) for ferromagnetism to prevail, as illustrated in Table 4. Additionally, attention is drawn to indirect exchange energy *Δ*_*x*_(pd), derived from the VB edge in down-spin (↓), which manifests as negative. This negative (−ve) value signifies the enhanced attractiveness of the down (↓) spin configuration for exchange mechanisms, thereby lowering the system's energy and affirming ferromagnetic stability. Despite the complexity of Mo's electronic configuration, d states of Mo engage in hybridization with the valence states of Tl and the 3p of Cl and 4p state of Br. Particularly, Mo's t_2g_ states exhibit robust hybridization with Cl's 3p state, with negligible influence from Tl's 6s-states, in energy ranges spanning from −0.24 eV to *E*_F_ and from −4.02 to −4.3 eV in the up (↑) spin configuration. Furthermore, a hybridization zone between the t_2g_ states of Mo and Cl's 3p states emerges in the −2.2 to −4.8 eV energy range although this range is not pertinent to our analysis.^41^ Notably, significant hybridization is observed between Mo's e_g_ and Cl's 3p states in the vicinity of the CB edge. In the down (↓) spin configuration, hybridization arises among Mo's t_2g_ and Cl's 3p states in VB and CB, respectively. However, the core region witnesses negligible participation in the hybridization process, as shown in Fig. 5. Similarly, robust hybridization is evident between Mo's t_2g_ and 4p states of Br, as illustrated in Fig. 6. Additionally, reasonable hybridization between Mo's e_g_ and Br's 4p states is observed in the conduction band. In the down (↓) spin configuration, hybridization arises among Mo's t_2g_, Br's 4p states, and Tl's 6s states in VB and CB. We also computed phonon dispersion curves for both investigated materials, revealing that no imaginary mode is observed for Tl_2_Mo(Cl/Br)_6_ (see Fig. 7). The positive value of the phonon frequency for both materials indicates that they are dynamically stable in the given temperature range.

**Table 4:** Calculated crystal field (Δ*C*_F_) energy, exchange splitting *Δ*_*x*_(d), and exchange constants (*N*_o_*α* and *N*_o_*β*) for Tl_2_Mo(Cl/Br)_6_

	Δ*C*_F_	*Δ*_*x*_(d)	*Δ*_*x*_(pd)	*N*_o_ *α*	*N*_o_ *β*
Tl_2_MoCl_6_	3.0	4.1	−1.5	0.38	−1.92
Tl_2_MoBr_6_	2.6	3.8	−1.2	0.25	−0.93

### 3.5 Magnetic properties

Understanding ferromagnetism relies significantly on the splitting of VB and CB edges, particularly concerning the interactions between s and d orbitals as well as p and d orbitals.^42^ These orbital interactions, denoted as s–d and p–d couplings, respectively, are delineated based on the average magnetic moments of Mo within the unit cell. The energy discrepancy between the up (↑) and down (↓) spin configurations at the CB edge (denoted as *E*_c_) predominantly reflects the influence of s–d coupling. Conversely, at the valence band edge, this energy difference (denoted as *E*_V_) encompasses contributions from p–d coupling. The specifics of these interactions are further expounded through the elucidation of exchange constants as follows:
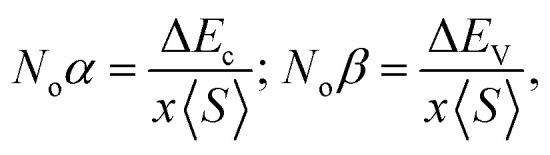
where *x* represents the concentration of Mo and 〈*S*〉 denotes its average magnetic moment.^43^ The presence of negative values for *N*_o_*β* indicates high level of attraction towards down (↓) spin configuration within the exchange mechanism, resembling the characteristics of ferromagnetism, akin to exchange energy *Δ*(pd), as outlined in Table 4. The transition of electrons from the down (↓) spin to up (↑) spin configuration, coupled with robust hybridization between the d states of Mo, 3p state of Cl, and Br 4p-states, introduces both orbital and spin magnetic moments, highlighting their significance in spintronic devices. Detailed magnetic moment data for individual elements Tl, Mo, and Cl/Br, as well as compounds Tl_2_Mo(Cl/Br)_6_, are provided in Table 5.^44^ The presence of magnetic moments on nonmagnetic elements arises from robust hybridization and SOC among the d state of Mo, 3p state of Cl and Br-4p state, underscoring the pivotal contribution of Mo's d state in spintronic applications. Furthermore, Table 5 displays both the total and individual magnetic moments for Tl_2_Mo(Cl/Br)_6_. The integral figures of total magnetic moments signify complete spin-polarization of the composition under investigation. The predominant source of magnetic moments stems from the Mo-d state. Strong hybridization particularly results in the transfer of the magnetic moments from the Mo site to Tl and Cl/Br.^45^

**Table 5:** Total and local values of magnetic moments for Tl_2_Mo(Cl/Br)_6_

	*M*_Total_	*M*_Int._	*M*_Tl_	*M*_Mo_	*M*_Cl/Br_
Tl_2_MoCl_6_	2.000	0.3489	−0.003	1.579	0.013
Tl_2_MoBr_6_	2.000	0.3824	−0.001	1.571	0.008

### 3.6 Thermoelectric properties

**Fig. 8:**
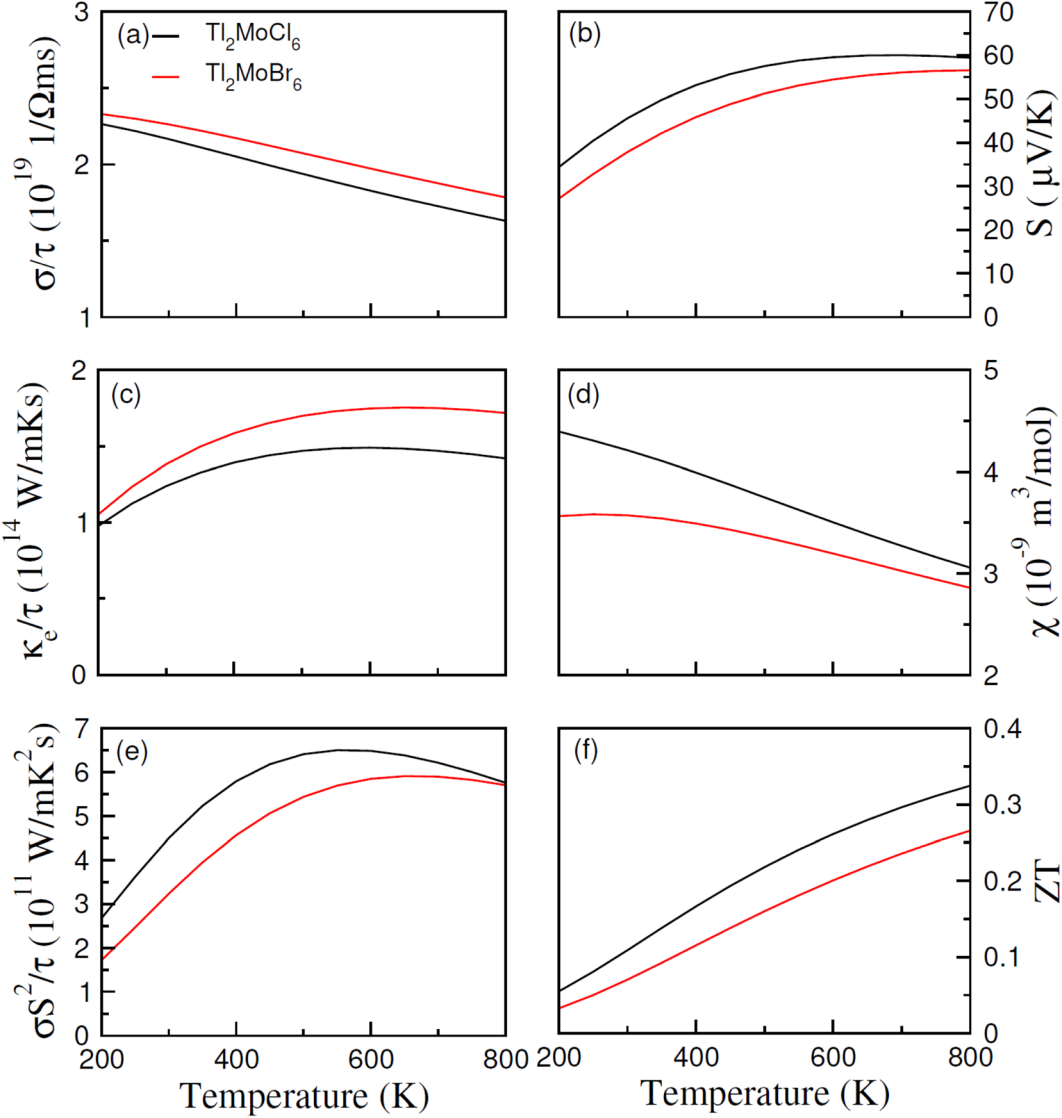
Calculated (a) electrical conductivity (*σ*/*τ*), (b) thermal conductivity (*k*_e_/*τ*), (c) Seebeck coefficients (*S*), (d) magnetic susceptibility (*χ*), (e) power factor and (f) *ZT versus* temperature for Tl_2_Mo(Cl/Br)_6_.

In recent years, noteworthy attention has been paid to the potential of a thermoelectric material to convert thermal energy into electrical energy across various applications.^46^ This phenomenon, known as the thermoelectric effect, relies on the transfer of charge movement to generate a heat gradient, thereby creating a potential difference. The thermoelectric properties of Tl_2_Mo(Cl/Br)_6_ were examined utilizing the BoltzTraP code with diverse parameters, as presented in Fig. 8(a–f). Within the BoltzTraP simulation, the relaxation time, set at 10^−14^ s, denotes the average duration between successive collisions within the system. Electrical conduction induced by charge carriers is determined by evaluating *σ*/*τ*.^47^ As depicted in Fig. 8(a), the transition from Cl to Br prompts a rapid escalation in *σ*/*τ*, which is attributable to the heightened presence of free electrons transitioning thermally from valence to the conduction band. At 200 K, *σ*/*τ* measures 2.5 × 10^19^ for Tl_2_MoCl_6_ and 2.7 × 10^19^ (Ω m s)^−1^ for Tl_2_MoBr_6_. Analogous trends persist at 800 K. The increase in conductivity following the substitution of Cl with Br can be ascribed to the larger ionic size and the augmented contribution of free carriers. However, as the temperature increases, conductivity decreases potentially due to heightened resistance experienced by free carriers amidst thermal agitation at higher temperatures. Thermal conductivity (*κ*_e_/*τ*) represents the carrier's conduction because of heat, with only the electronic component calculated while disregarding lattice contributions. For Tl_2_MoCl_6_, thermal conductivity escalates from 1.0 × 10^14^ at 200 K to 1.42 × 10^14^ (W m^−1^ K^−1^ s^−1^) at 800 K, and a similar trend is observed for Tl_2_MoBr_6_, as illustrated in Fig. 8(c). The optimal performance is indicated by minimizing *κ*_e_/*τ*, as observed from *κ*/*σ*. The electronic to thermal (*κ*/*σ*) conductivity ratio is in the order of 10^−6^,^48^ emphasizing their significance for thermoelectric applications due to their lower *κ*/*τ* values related to *σ*/*τ*. The Seebeck coefficient (*S*) serves as a crucial gauge of potential gradients and is charted against the temperature. The polarity of *S* values, negative or positive, delineates whether electrons or holes predominate as charge carriers. For temperatures ranging from 200 to 800 K, Fig. 8(c) illustrates the fluctuation in *S*. Notably, for Tl_2_Mo(Cl/Br)_6_, the *S* value increases as the temperature increases potentially due to disparities in energy magnitude among the upper VB and *E*_f_,^49^ as illustrated in Fig. 8(c). The magnetic response of materials is characterized by their susceptibility, typically around 10^−9^ for semiconductors. At 200 K, Tl_2_Mo(Cl/Br)_6_ exhibits susceptibility of 4.4 × 10^−9^ and 3.6 × 10^−9^ m^3^ mol^−1^, respectively; then, its value decreases at 800 K, which is potentially influenced by thermal effects on electron spin movement, as depicted in Fig. 8(d). In Fig. 8(d), *σS*^2^/*τ*, termed the power factor (PF), representing the thermoelectric potency of materials, is drawn against the temperature. The behavior of *σ*/*τ* and PF aligns closely due to the extremely conductive response of a material. As the temperature increases, PF diminishes upon substituting Cl with Br.^50^ Comparative analysis reveals that PF for Tl_2_MoCl_6_ surpasses that of Tl_2_MoBr_6_ due to the predominance of n-type carrier contributions over p-type carriers.^51^ The figure of merit (*ZT*), illustrated in Fig. 8(f), is expressed by *ZT* = *S*^2^*σ*/*κT*. A high *ZT* value at low-temperature values indicates minimal thermal conductivity alongside a small *S* and higher *σ*/*τ*. The relationship for *ZT* demonstrates a direct proportional trend between *κ*_e_/*τ* and *σ*/*τ* against temperature. The curve for Tl_2_MoBr_6_ is lower, attributed to slightly greater *S* and high *κ*_e_/*τ* compared to the Tl_2_MoCl_6_ curve. The maximum *ZT* values are 0.33 for Tl_2_MoCl_6_ and 0.26 for Tl_2_MoBr_6_ at 800 K.^52^

## 4. Conclusion

In the current study, a thorough analysis of the half metallic ferromagnetic and thermoelectric responses of Tl_2_Mo(Cl/Br)_6_ double perovskites was conducted to explore their perspectives in spintronics and thermoelectric applications. Initially, structural optimization was performed in the ferromagnetic (FM) and anti-ferromagnetic (AFM) phases, confirming the stability of the FM phase. Additionally, the formation energies of Tl_2_MoCl_6_ and Tl_2_MoBr_6_ are −1.44 and −1.88 eV, respectively, indicating thermodynamic stability. The 100% spin polarization was confirmed through spin-polarized electronic DOS, revealing underlying hybridization and fractional magnetic moments alongside higher *T*_c_. Exchange energies and valence electron hybridization further confirmed the ferromagnetic response attributed to electron spin instead of clustering. Quantum confinement predominantly influenced negative exchange coefficient values and p–d exchange energy. Moreover, the calculations of *S* unveiled the existence of p-type semiconducting characteristics in Tl_2_Mo(Cl/Br)_6_. The notably higher *ZT* at 800 K observed as 0.33 and 0.26 for Tl_2_MoCl_6_ and Tl_2_MoBr_6_, respectively, suggested their comparative suitability for thermoelectric device application.

## Data availability

All data presented in this manuscript can be provided by the corresponding author on reasonable demand.

## Conflicts of interest

The authors declare that they have no known competing financial interests or personal relationships that could have appeared to influence the work reported in this paper.

## Supplementary Material
